# Alcohols enhance the rate of acetic acid diffusion in *S. cerevisiae*: biophysical mechanisms and implications for acetic acid tolerance

**DOI:** 10.15698/mic2018.01.609

**Published:** 2017-12-01

**Authors:** Lina Lindahl, Samuel Genheden, Fábio Faria-Oliveira, Stefan Allard, Leif A. Eriksson, Lisbeth Olsson, Maurizio Bettiga

**Affiliations:** 1 Department of Biology and Biological Engineering, Division of Industrial Biotechnology, Chalmers University of Technology, Gothenburg, Sweden.; 2 Department of Chemistry and Molecular Biology, University of Gothenburg, Gothenburg, Sweden.; 3 Department of Chemistry and Chemical Engineering, Division of Energy and Materials, Nuclear Chemistry, Chalmers University of Technology, Gothenburg, Sweden.; 4 EviKrets Biobased Processes Consultants, Gothenburg, Sweden.

**Keywords:** ethanol, n-butanol, lignocellulose, inhibitors, molecular dynamics simulations, membrane permeation, carbon-14 uptake

## Abstract

Microbial cell factories with the ability to maintain high productivity in the presence of weak organic acids, such as acetic acid, are required in many industrial processes. For example, fermentation media derived from lignocellulosic biomass are rich in acetic acid and other weak acids. The rate of diffusional entry of acetic acid is one parameter determining the ability of microorganisms to tolerance the acid. The present study demonstrates that the rate of acetic acid diffusion in *S. cerevisiae* is strongly affected by the alcohols ethanol and n-butanol. Ethanol of 40 g/L and n-butanol of 8 g/L both caused a 65% increase in the rate of acetic acid diffusion, and higher alcohol concentrations caused even greater increases. Molecular dynamics simulations of membrane dynamics in the presence of alcohols demonstrated that the partitioning of alcohols to the head group region of the lipid bilayer causes a considerable increase in the membrane area, together with reduced membrane thickness and lipid order. These changes in physiochemical membrane properties lead to an increased number of water molecules in the membrane interior, providing biophysical mechanisms for the alcohol-induced increase in acetic acid diffusion rate. n-butanol affected *S. cerevisiae* and the cell membrane properties at lower concentrations than ethanol, due to greater and deeper partitioning in the membrane. This study demonstrates that the rate of acetic acid diffusion can be strongly affected by compounds that partition into the cell membrane, and highlights the need for considering interaction effects between compounds in the design of microbial processes.

## INTRODUCTION

Weak organic acids, such as acetic acid, are inhibitory to many microorganisms 1,2. The rate of acetic acid diffusion into the cell is one of the parameters influencing the microorganism’s tolerance. Two modes of acetic acid entry have been described in the yeast *Saccharomyces cerevisiae* under respiro-fermentative growth on glucose: passive diffusion and facilitated diffusion. Passive diffusion across the lipid bilayer of the cell membrane has traditionally been described as the major mode of entry of acetic acid in *S. cerevisiae* 3, while facilitated diffusion through the aquaglyceroporin Fps1 was more recently reported to be a possible mode of entry of acetic acid, under certain conditions 4. The molecular species entering the cell is the more hydrophobic undissociated (protonated) form of acetic acid (pKa 4.8) for both modes of entry. Once inside the cell, acetic acid dissociates, as the cytosolic pH of *S. cerevisiae* is close to neutral 5. As a result of the dissociation of acetic acid inside the cell, more acetic acid diffuses in until equilibrium is reached between the undissociated acetic acid on both sides of the cell membrane. As a consequence, acetate and protons accumulate inside the cell 6, and the concentration at equilibrium is often high, due to a commonly higher intracellular than extra-cellular pH. The major challenge for *S. cerevisiae* is to counteract this accumulation by ATP-dependent acetate and proton efflux 7,8,9. If the cell succeeds in balancing the rate of acetic acid diffusion with the rate of acetate and proton efflux, cell growth will continue 10. If the diffusion rate is higher than the removal capacity, acetate and protons will accumulate in the cells, and they will be unable to continue growing. This need for a balance explains why the rate of acetic acid diffusion by influencing the intracellular acetic acid accumulation also affects the concentration of acetic acid that allows cell growth.

Knowledge on the mechanisms controlling the effect of acetic acid on cell growth and physiology is important in several applications. When using lignocellulosic biomass as the raw material for biofuel and biochemical production, acetylated hemicellulose releases acetic acid upon pre¬treatment of the material 11. The exact acetic acid concentration depends on the source of lignocellulose and the pretreatment method, but is commonly in the range 5 - 10 g/L 11,12, affecting the cell physiology of common strains of *S. cerevisiae* 13,14. Furthermore, weak acid tolerance of microorganisms plays an important role in the use of weak acids in food preservation 9, in the microbial production of weak acids 15, and it also affects the tolerance of microorganisms to all other chemicals that exerts an inhibitory, synergistic effect with the weak acids, as investigated by the present work.

Some of the physiological mechanisms of cell inhibition by acetic acid have been extensively studied and reviewed 1,2,16. Acetate and proton efflux are energy dependent, resulting in reduced cellular pools of ATP 17. Under conditions of low intracellular ATP consumption for growth and maintenance, surplus ATP may be sufficient, while at higher consumption rates increased ATP consumption for maintenance (including acetate and proton efflux) is reflected by lower maximum specific growth rate 13 and lower biomass yield. Intracellular acetate and proton accumulation cause a range of cellular effects such as reduced enzyme activity 18,19, changes in membrane potential 5, and the formation of reactive oxygen species (ROS), which if severe, leads to apoptosis 20. Factors influencing the rate of acetic acid diffusion into the cell have been investigated to a lesser extent. We have previously demonstrated that exposure to acetic acid causes rearrangement of the membrane lipid profile in *S. cerevisiae*, possibly resulting in a reduction in the acetic acid diffusion rate, although the change in lipid profile was not as extensive as in the highly acetic-acid-tolerant yeast *Zygosaccharomyces bailii* 13. We have also shown that the membrane lipid composition high in sphingolipids is important in *Z. bailii* for its low acetic acid diffusion rate, and its high acetic acid tolerance 10. Furthermore, it has been demonstrated that cells preadapted to acetic acid exhibit a slower rate of acetic acid entry than non-adapted cells 17. Changes in the membrane lipid profile have also been shown to increase acetic acid tolerance in *S. cerevisiae* 21, probably through a reduction in the acetic acid diffusion rate.

As membrane lipid composition and physico-chemical membrane properties influence the rate of acetic acid diffusion, we hypothesized that compounds that partition into the membrane also influence the rate of acetic acid diffusion. To test this hypothesis, we used the alcohols ethanol and n-butanol, as they have been demonstrated to partition into the membrane and affect the membrane properties 22,23. Studying the synergistic effect between alcohols and acetic acid is also relevant as ethanol and n-butanol are two chemicals likely to be produced from lignocellulose 24,25. The main subject of this study was the investigation of the extent to which alcohols affect the rate of acetic acid diffusion. We addressed this question experimentally by measuring the diffusion of ^14^C-labeled acetic acid into *S. cerevisiae*. A computational approach, using molecular dynamics simulations, was then used to simulate the effect of alcohols on a complex model membrane, designed to resemble the membrane lipid composition of *S. cerevisiae*, in order to elucidate the biophysical mechanisms responsible for the observed effect of alcohols on the diffusion rate. Furthermore, we investigated how an increase in the rate of acetic acid diffusion, induced by alcohols, affected the cells’ tolerance to acetic acid, by evaluating the combined effect of acetic acid and ethanol or n-butanol on the specific growth rate of *S. cerevisiae*.

## RESULTS

The effect of alcohols on the acetic acid diffusion rate was investigated experimentally in *S.* *cerevisiae* using concentrations of alcohols that allowed cell growth. For this reason, higher ethanol concentrations were compared with lower n-butanol concentrations. The implications of changes in the acetic acid diffusion rate were then investigated on cells grown in the presence of alcohol and acetic acid. Furthermore, molecular dynamics simulations were used to simulate the effect of alcohols on a model membrane to elucidate the biophysical mechanisms determining the rate of acetic acid diffusion through the lipid bilayer of the cell membrane. As this approach was adopted to elucidate mechanisms and to observe trends, equal concentrations of ethanol and n butanol were compared.

### The effect of acetic acid concentration and the contribution of Fps1 to the rate of acetic acid diffusion in *S. cerevisiae*

**Figure 1:**
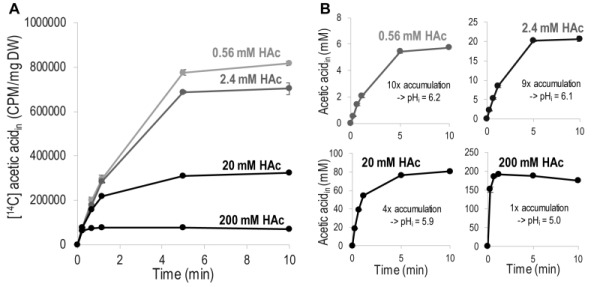
FIGURE 1: Diffusion of acetic acid (HAc) into *S. cerevisiae* as a function of time. Diffusion was measured in phthalate buffer at pH 5 using 1 µCi [^14^C] acetic acid mixed with non-labeled acetic acid to the indicated final concentrations. **(A)** Acetic acid accumulation showing only the signal from [^14^C] acetic acid. **(B)** Accumulation of total (labeled + unlabeled) acetic acid inside the cell. Calculated from the [^14^C] acetic acid signal assuming a cell volume of 2 µL/mg dry weight. The indicated magnitude of accumulation is given by the maximum concentration of intracellular acetic acid divided by the concentration of acetic acid added to the diffusion assay. The amount of acetic acid accumulated was then converted into intracellular pH values (pH_i_) using the Henderson-Hasselbalch equation. The data given are the mean of three biological replicates, and the error bars indicate the standard deviation. Abbreviations; DW, cell dry weight; CPM, counts of ^14^C decay per minute.

Acetic acid diffusion is preferably quantified by its initial rate, before interference by accumulated intracellular acetic acid. To determine the concentration and time interval allowing quantification of the initial rate of acetic acid diffusion, the increase in intracellular concentration of acetic acid in cells exposed to 0.56 - 200 mM acetic acid was monitored for 10 minutes (Figure 1). To facilitate comparison, acetic acid at each concentration was mixed with a constant amount of [^14^C] acetic acid, and the signal from only [^14^C] acetic acid is shown in Figure 1A. The curves in Figure 1A show the same initial slope, which demonstrates that the initial rate of acetic acid diffusion, per molecule, is independent of the total concentration of acetic acid, at least up to 200 mM. However, the time required to reach equilibrium between the acetic acid inside and outside the cell is shorter with increasing concentration of acetic acid, resulting in the entry of fewer labeled acetic acid molecules at the higher total concentrations evaluated (Figure 1A). These findings demonstrate that relatively low acetic acid concentrations must be used to determine diffusion kinetics, unless a very rapid sampling procedure is available. The initial slope of the acetic acid diffusion rate for all the acetic acid concentrations evaluated further indicates that acetic acid itself does not affect the cell membrane to change the rate of acetic acid diffusion.

An indication of the magnitude of intracellular acetic acid accumulation was inferred by recalculating the [^14^C] acetic acid with respect to the total concentration of intracellular acetic acid (Figure 1B); for the calculations, a cell volume of 2 µL/mg dry weight was assumed 42, over the period of 10 minutes investigated, 0.2 mM extracellular acetic acid resulted in a ten-fold accumulation inside the cell, while 2 mM resulted in a nine-fold accumulation, and 20 mM resulted in a four-fold accumulation. Extracellular acetic acid at a concentration of 200 mM resulted in similar concen¬trations of extracellular and intracellular acetic acid. Intracellular accumulation can be used to estimate the intracellular pH (pH_i_) by using the Henderson-Hasselbalch equation 26. Acetic acid at concentrations of 0.2 mM and 2 mM affected intracellular pH to a minor extent, resulting in pH_i_ values of 6.2 and 6.1, respectively. Acetic acid at a concentration of 20 mM reduced pH_i_ to 5.9, while 200 mM acetic acid reduced it to 5.0, i.e., the same as the extracellular pH. Thus, it is evident that increasing the concentration of extracellular acetic acid increases the concentration of intracellular acetic acid, but as intracellular acetic acid affects the intracellular pH, this results in a concentration-dependent decrease in the relative intracellular acetic acid accumulation.

**Figure 2:**
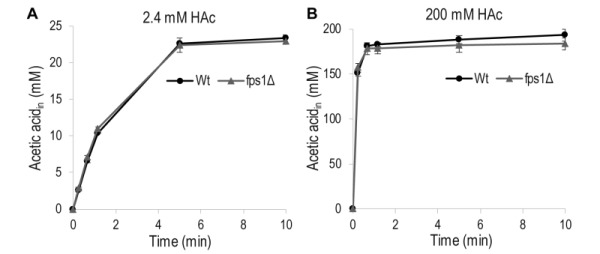
FIGURE 2: Accumulation of acetic acid (HAc) in wild-type (wt) *S. cerevisiae* cells and cells lacking the aquaglyceroporin Fps1 (*fps1*Δ) as a function of time. Diffusion was measured in phthalate buffer at pH 5 using 1 µCi [^14^C] acetic acid mixed with non-labeled acetic acid to the indicated final concentrations. Accumulation of total (labeled + unlabeled) acetic acid inside the cell when **(A)** 2.4 mM acetic acid was added, and **(B)** when 200 mM acetic acid was added. The data given are the mean of three biological replicates, and the error bars indicate the standard deviation.

To determine the extent to which acetic acid entry occurs through Fps1, as opposed to passive diffusion through the lipid bilayer of the cell membrane, in our experimental setup, the acetic acid diffusion rate was measured in wild-type *S. cerevisiae* cells and a mutant strain, *fps1*Δ, lacking Fps1. No significant differences were observed between the rates of acetic acid diffusion in the wild-type and mutant cells, when they were exposed to a low (2 mM) or a high (200 mM) concentration of acetic acid (Figure 2). This demonstrates that acetic acid diffusion is not influenced by Fps1 under our experimental conditions, and passive diffusion of acetic acid across the lipid bilayer is probably the main mode of entry.

### The effect of alcohols on the rate of acetic acid diffusion in* S. cerevisiae*

**Figure 3:**
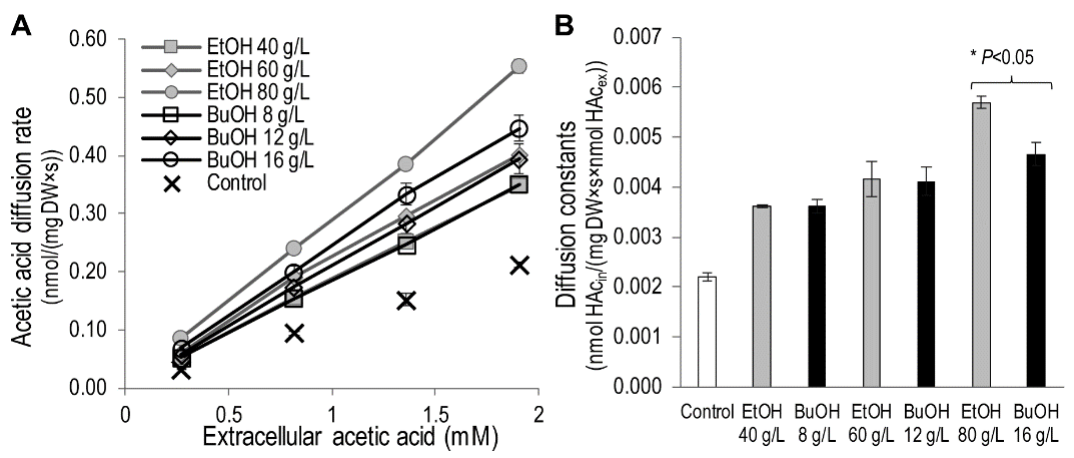
FIGURE 3:Kinetics of the acetic acid diffusion rate in *S. cerevisiae* in response to ethanol concentrations of 40 - 80 g/L and n butanol concentrations of 8 - 16 g/L. **(A) **Acetic acid diffusion rate as a function of extracellular acetic acid. **(B)** Diffusion constants calculated from the slope of the kinetics curves presented in A, although the number of moles of extracellular acetic acid was plotted on the x-axis instead of the molar concentration. The data given are the means of three biological replicates, and the error bars indicate the standard deviation. *Statistically significant difference according to the t-test.

To investigate the effect of ethanol and n butanol on the acetic acid diffusion rate, acetic acid diffusion kinetics were determined in the presence of the two alcohols by measuring the intracellular acetic acid concentration 30 seconds after exposing the cells to acetic acid at concentrations of 0.2 - 1.4 mM (Figure 3). Ethanol concentrations in the range 40 - 80 g/L and n-butanol concentrations in the range 8 - 16 g/L were evaluated. Both the lowest concentrations evaluated (40 g/L ethanol and 8 g/L n-butanol) resulted in an increase in the acetic acid diffusion rate of approximately 65% compared to the control. Moreover, both 60 g/L ethanol and 12 g/L n butanol resulted in an approximately 90% increase in the acetic acid diffusion rate compared to the control. At higher alcohol concentrations (80 g/L ethanol and 16 g/L n-butanol), the effect of ethanol was more severe, causing an approximately 160% increase in the acetic acid diffusion rate, while n-butanol exposure caused a 115% increase. It can therefore be concluded that ethanol and n butanol greatly increase the rate of acetic acid diffusion in *S. cerevisiae*.

### The effect of alcohols and acetic acid on the growth of *S. cerevisiae*

To elucidate the implications of the increase in the acetic diffusion rate, due to alcohols, on acetic acid tolerance, cell growth was evaluated in the presence of alcohols and acetic acid. Cell growth was evaluated quantitatively in terms of the maximum specific growth rate and the time required to consume the available glucose. The latter term was introduced to define the growth of strongly inhibited cells that did not exhibit a clear exponential growth profile, and exhibits a possible lag phase of the cells before growth was resumed. Interestingly, 40 g/L ethanol and 8 g/L n butanol, which resulted in similar increases in the acetic acid diffusion rate, also affected the maximum specific growth rate and the time required to consume the available glucose to a similar degree (Table 1). Furthermore, 60 g/L ethanol and 12 g/L n butanol, which also resulted in similar increases in the acetic acid diffusion rate, also caused the cells to consume the available glucose in relatively similar times of 34 hours and 40 hours, respectively. The maximum specific growth rates with these alcohol concentrations could not be compared due to the non-exponential growth of cells in 12 g/L n butanol. No growth was observed when *S. cerevisiae* was cultured in 80 g/L ethanol or 16 g/L n butanol.

**Table 1:** The effect of ethanol and n hyphen butanol on growth of *S. cerevisiae* in the absence and presence of acetic acid. Cells were cultured aerobically in minimal medium at pH 5, supplemented with the indicated concentrations of ethanol, n butanol and acetic acid. Values given are the mean of three biological replicates ± standard deviation.

**Condition**	**Maximum specific growth rate (h^-1^)**	**Time to consume glucose (h)**
*Alcohol only*		
Control	0.35 ± 0.00	7.7 ± 0.1
EtOH, 40 g/L	0.15 ± 0.01	15 ± 0
BuOH, 8 g/L	0.12 ± 0.01	14 ± 1
EtOH, 60 g/L	0.04 ± 0.02	34 ± 1
BuOH, 12 g/L	Non-exponential growth	40 ± 9
EtOH, 80 g/L	No growth	No growth in 70 hours
BuOH, 16 g/L	No growth	No growth in 70 hours
*Acetic acid (3 g/L) and alcohol*		
Acetic acid only	0.34 ± 0.01	8.1 ± 0.1
EtOH, 40 g/L	0.09 ± 0.01	18 ± 0
BuOH, 8 g/L	0.11 ± 0.01	14 ± 1
*Acetic acid (6 g/L) and alcohol*		
Acetic acid only	0.30 ± 0.00	9.1 ± 0.0
EtOH, 40 g/L	No growth	No growth in 70 hours
BuOH, 8 g/L	0.05 ± 0.00	44 ± 4

To obtain an indication on how the increase in the acetic acid diffusion rate caused by alcohols affects the cells’ tolerance to acetic acid, the combined effect of acetic acid and ethanol or n-butanol on cell growth was evaluated. The effect of alcohols in the presence of acetic acid was first evaluated using a relatively low acetic acid concentration of 3 g/L. This had no effect on the maximum specific growth rate or the time required to consume the glucose (Table 1), but energy appeared to be used for acetate and proton efflux, as indicated by a prolonged lag phase after glucose depletion, before switching to respiratory growth (data not shown). This delay in resuming growth after the diauxic shift was probably due to insufficient ATP to avoid uncontrolled accumulation of acetic acid inside the cell, when shifting metabolism. Thus, cells grown in medium containing alcohol and acetic acid (with an increased acetic acid diffusion rate caused by ethanol and n butanol) could potentially have a higher specific ATP consumption rate, due to acetate and proton efflux, than cells grown in medium with only acetic acid. Consequently, cells exposed to 40 g/L ethanol and 3 g/L acetic acid showed a greater reduction in maximum specific growth rate, and a greater increase in the time required to consume the glucose, than cells exposed to ethanol only (Table 1). However, the growth of cells exposed to 8 g/L n-butanol and 3 g/L acetic acid was not affected more severely than that of cells exposed to n butanol only (Table 1). This suggests that cells exposed to 8 g/L n-butanol and 3 g/L acetic acid still have surplus ATP available to handle the increase in energy demand caused by the acetic acid addition. However, this speculation needs to be confirmed in a separate study under a dedicated experimental setup. The greater growth reduction following exposure to 40 g/L ethanol than 8 g/L n butanol, when combined with 3 g/L acetic acid, may thus be due to intracellular effects not related to the properties of the cell membrane.

The effect of alcohols on cell growth in the presence of acetic acid, was evaluated using a higher acetic acid concentration, 6 g/L, which alone caused a 15% reduction in the maximum specific growth rate and a 15% increase in the time required to consume all the available glucose, compared with the control (Table 1). At this higher acetic acid concentration, it is difficult to distinguish growth reduction due to alcohol exposure from growth reduction due to acetic acid exposure, but it is again evident that cell growth in the presence of 40 g/L ethanol is more affected by the addition of acetic acid than 8 g/L n-butanol (Table 1). *S. cerevisiae* cells exposed to acetic acid and ethanol were unable to resume growth within 70 hours, while cells exposed to acetic acid and n-butanol grew slowly after a long lag phase, and consumed the available glucose in 44 hours.

### Simulations of alcohol partitioning in the membrane

A computational approach was adopted using molecular dynamics simulations to elucidate possible biophysical mechanisms behind the observed increase in acetic acid diffusion rate caused by ethanol and n-butanol. The simulated model membrane was designed to resemble the membrane lipid composition of the cell membrane in *S*. *cerevisiae*, and included the glycerophospholipids DOPC (1,2-dioleoyl-sn-glycero-3-phosphocholine) and POPI (1-palmitoyl-2-oleoyl-sn-glycero-3-phosphoinositol), the sphingolipid IPC (inositol phosphorylceramide) and the sterol ergosterol 10. In the simulations, alcohols were added to the water phase at the indicated concentrations, and the reported data were extracted after equilibrium was reached between the water phase and the membrane, each occupying approximately 50% of the simulation box. Alcohol concentrations used in our experimental setup should not be directly compared with the simulated alcohol concentrations due to differences in the two systems, mainly with respect to the volume of the water phase.

**Figure 4:**
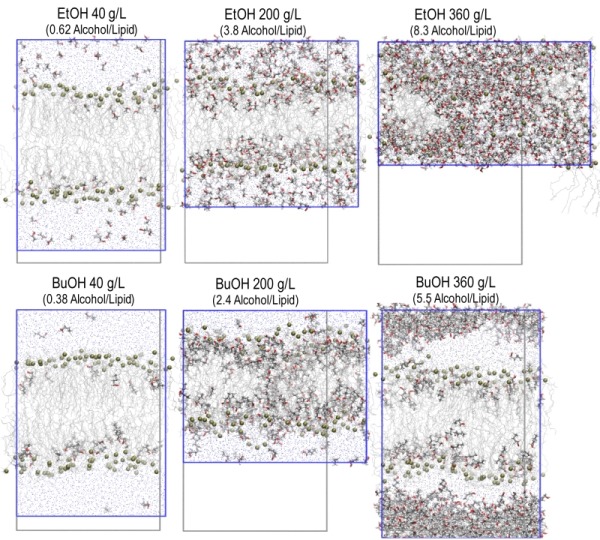
FIGURE 4: Snapshots from simulations with 40, 200, and 360 g/L of ethanol and n-butanol. Alcohol molecules are shown in gray with the hydroxyl group in red. The water and lipids are shown as thin gray lines with the phosphate groups on the lipids marked with green balls. The simulation box is indicated in blue, and the simulation box at 8 g/L alcohol is indicated in gray.

From the snapshots from the simulations, shown in Figure 4, it can be seen that both ethanol and n butanol partition into the membrane. Alcohol concentrations of 40 g/L have a minor impact on the membrane appearance in the simulations, while 200 g/L ethanol drastically changes the appearance of the membrane, although the bilayer structure is still intact. At the corresponding concentration of n butanol, pores are formed in the membrane. Ethanol at a concentration of 360 g/L completely disrupts the membrane, while the same concentration of n-butanol only affects the membrane to a minor extent, due to the formation of a butanol phase outside the membrane. The simulations of alcohols at concentrations of 360 g/L will therefore not be further discussed.

**Figure 5:**
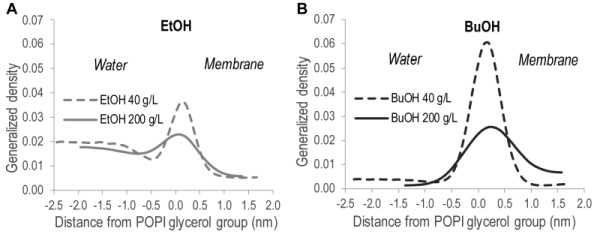
FIGURE 5: Location of the hydroxyl group of alcohols along the membrane normal relative to the glycerol backbone of the POPI lipid. X = 0 corresponds to the peak location of the glycerol backbone of the POPI lipid. Distances < 0 indicate the water phase, while distances > 0 indicate the lipid bilayer. **(A)** Ethanol. **(B)** n-butanol. The data shown are the mean values over one simulation ± standard deviation.

In order to quantitatively illustrate alcohol localization, the density of alcohols through the membrane was plotted. The glycerol group of POPI was defined to be at zero, the water phase at negative values, and the membrane interior at positive values (Figure 5). The density plots illustrate that both ethanol and n butanol preferably partition in the head group region of the lipid bilayer close to the glycerol backbone of the POPI lipids, thus providing a first indication of why alcohols affect the acetic acid diffusion rate in *S. cerevisiae*. However, a significant fraction of ethanol also partitions to the water phase, while very little of the n butanol is found in the water phase (Figure 5, Table 2). When averaged over the simulations with different initial alcohol concentrations, 59 ± 4% and 94 ± 5% of the ethanol and n butanol molecules, respectively, are found in the membrane. Furthermore, the distribution of alcohols in the membrane depends on their specific concentration. At low concentrations, the distribution of the alcohols is narrow, while at higher concentrations the distribution is broader (Figure 5). At high alcohol concentration, a fraction of the n-butanol molecules is seen deep in the membrane (Figure 5), which indicates the formation of a pore in the membrane. Indeed, a pore was observed in the snapshot from the simulation with 200 g/L n-butanol (Figure 4). The density plots in Figure 5 indicate the position of the hydroxyl group of the two alcohols, showing no significant difference between ethanol and n butanol. However, when the terminal methyl group of the two alcohols is compared, it is clear that n-butanol partitions deeper in the membrane than ethanol (Table 2). The methyl group of ethanol partitions approximately 0.20 nm deeper in the membrane than the glycerol backbone of the POPI lipid (Table 2), and there appears to be no preferred orientation of the ethanol molecule in the membrane (data not shown). The terminal methyl group of n butanol partitions approximately 0.40 - 0.50 nm deeper in the membrane than the glycerol backbone of the POPI lipid, depending on the specific concentration evaluated (Table 2). No preferred orientation of the hydroxyl group of n butanol was seen, but the terminal methyl group partitions deeper in the membrane than the hydroxyl group at all concentrations (data not shown). The deeper partitioning of n-butanol than ethanol is a direct consequence of the greater length of the n butanol molecule. According to the simulations, the ethanol molecule measures 0.15 nm from carbon 1 to carbon 2, while the n butanol molecule measures 0.37 nm from carbon 1 to carbon 4. The stronger effect of n butanol than ethanol on the acetic acid diffusion rate can thus be explained biophysically by the greater number of n butanol molecules partitioning into the membrane, and by partitioning deeper in the membrane than ethanol (Table 2).

**Table 2:** Partitioning of ethanol and n-butanol in the membrane at the simulated alcohol concentrations. ^1 ^The total amount of alcohols added to the simulation divided by the number of simulated lipids. ^2^ Depth of the terminal methyl group of ethanol and n-butanol in the membrane. Values given are the mean over one simulation ± standard deviation.

**Initial alcohol conc. (g/L)**	**Alcohol/Lipid^1^**		**Membrane partitioning (%)**		**Membrane depth (nm)^2^**	
	**EtOH**	**BuOH**	**EtOH**	**BuOH**	**EtOH**	**BuOH**
8	0.13	0.08	61 ± 1	84 ± 0	0.21 ± 0.09	0.31 ± 0.10
12	0.19	0.13	56 ± 1	99 ± 0	0.21 ± 0.07	0.40 ± 0.09
16	0.25	0.16	57 ± 0	98 ± 0	0.10 ± 0.09	0.40 ± 0.09
24	0.38	0.24	56 ± 1	90 ± 0	0.21 ± 0.08	0.52 ± 0.09
40	0.62	0.38	54 ± 1	89 ± 0	0.20 ± 0.07	0.51 ± 0.09
60	0.93	0.62	58 ± 1	95 ± 0	0.21 ± 0.10	0.50 ± 0.08
80	1.4	0.84	62 ± 1	96 ± 0	0.21 ± 0.08	0.49 ± 0.09
120	2.1	1.3	59 ± 1	95 ± 0	0.21 ± 0.08	0.41 ± 0.10
200	3.8	2.4	65 ± 1	97 ± 0	0.20 ± 0.09	0.42 ± 0.12

### Effect of alcohols on the physiochemical properties of the model membrane

**Figure 6:**
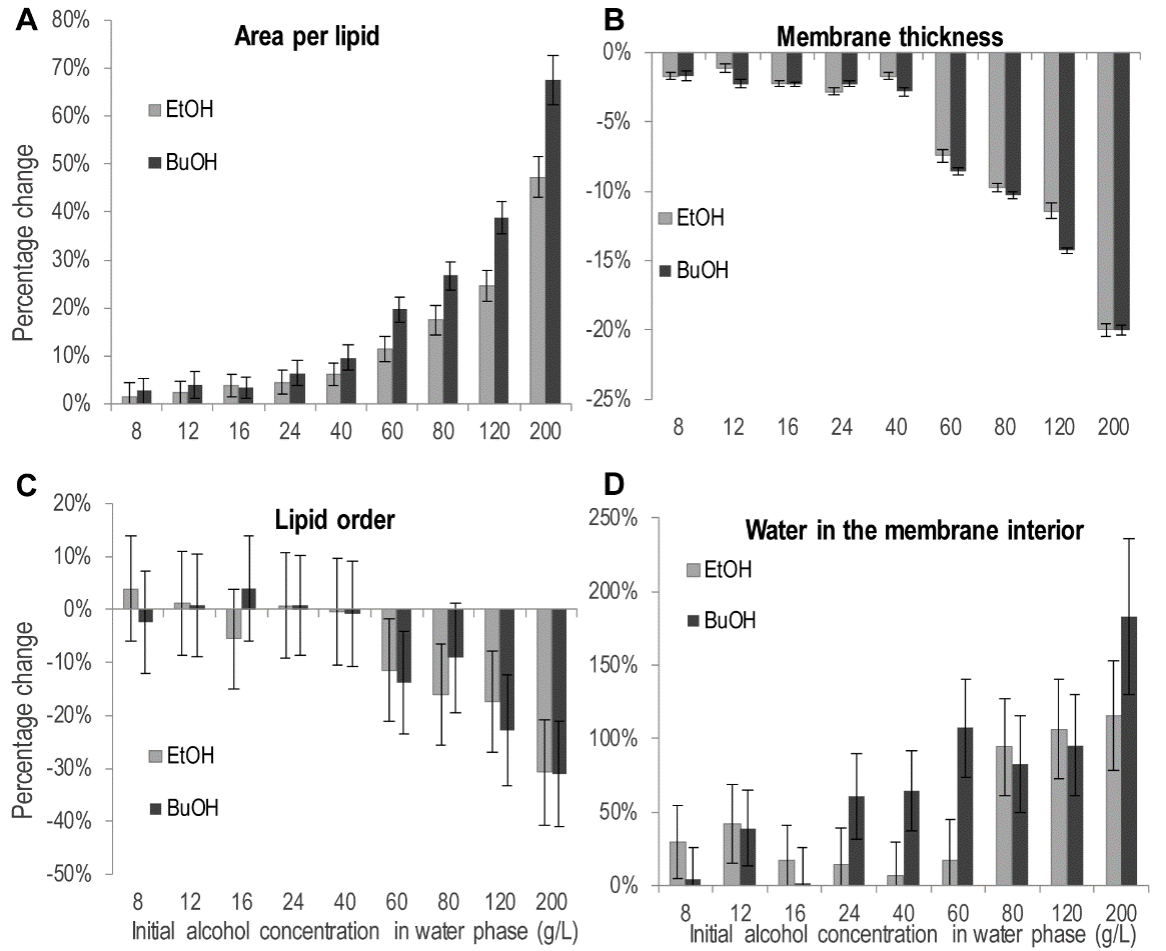
FIGURE 6: The effect of ethanol and n butanol on physiochemical membrane properties. **(A)** Area per lipid. **(B)** Membrane thickness. **(C)** Lipid order, calculated from the deuterium order parameter on the short chain of the IPC lipid. **(D)** Water in the membrane interior, defined as the number of water molecules in the lipid tail section of the bilayer. Percentage increase or decrease compared to the simulation without alcohol. The data shown are the mean over one simulation ± standard deviation.

Partitioning of ethanol and n-butanol in the head group region of the lipid bilayer influenced the physiochemical properties of the membrane (Figure 6). The area per lipid, i.e. the area of the simulation box in the membrane plane divided by the number of lipids, increased upon the addition of both alcohols (Figure 6A). The increase was significant for 40 g/L ethanol and 24 g/L n-butanol, and above. Considering the increased membrane partitioning of n butanol compared to ethanol, and the higher molecular weight of n-butanol, 40 g/L alcohol in the simulations corresponds to the partitioning of 42 ethanol molecules and 43 butanol molecules in the membrane, respectively. Hence differences in the physiochemical membrane properties caused by ethanol and n butanol presented in Figure 6 depend only on the deeper partitioning of the n butanol molecule in the membrane. Therefore, the difference in area per lipid between ethanol and n-butanol presented in Figure 6A, indicates that the deeper membrane partitioning of the n-butanol molecule further increases the area per lipid. For instance, 200 g/L of ethanol increased the area per lipid by 49%, from 0.53 to 0.79 nm^2^, while 200 g/L of n-butanol increased the area per lipid by 68%, from 0.53 to 0.89 nm^2^. The addition of alcohols also decreased the membrane thickness, but the magnitude was relatively small, and the difference between the effects of ethanol and n-butanol was small (Figure 6B). An increase in membrane area and a decrease in membrane thickness would probably result in a more fluid membrane. Membrane fluidity can be estimated from the deuterium order parameter, which provides a measure of lipid order; a low lipid order corresponding to high membrane fluidity. The average lipid order calculated from the deuterium order parameter of each carbon on the short chain of the IPC sphingolipid was affected by both alcohols, but only at the highest concentrations evaluated (Figure 6C). Due to large standard deviations in the lipid order measurements no clear difference could be seen between the effects of ethanol and n-butanol. When evaluating the lipid order carbon by carbon, the difference in lipid order was greatest around the middle of the fatty acyl chains (Figure S1).

The increased number of water molecules in the membrane interior observed during the simulations is a clear indication that alcohols make the membrane less dense, and probably explains the experimentally observed increase in acetic acid diffusion rate caused by alcohols (Figure 6D). The number of water molecules in the membrane interior was significantly higher at 80 g/L ethanol and above, while it was significantly higher already at 24 g/L n-butanol. This demonstrates the stronger effect of n butanol than ethanol, and the difference between alcohols is especially large at alcohol concentrations of 60 g/L and below. For example, in the membrane simulation without alcohols, 110 water molecules were present in the membrane interior, while at 40 g/L of each alcohol, there were only 7 more water molecules in the membrane interior for ethanol than without alcohol addition, while for n-butanol there were 71 more water molecules, corr-esponding to more than one alcohol per two membrane lipids.

## DISCUSSION

The rate of acetic acid diffusion into the cell influences the ability of the cells to tolerate and proliferate in the presence of this weak organic acid 9,10. In the present study, we demonstrated that the rate of acetic acid diffusion in *S. cerevisiae*, and thus its effects on the cell, is strongly affected by the two alcohols ethanol and n butanol. Ethanol at a concentration of 40 g/L and n-butanol at a concentration of 8 g/L both increased the acetic acid diffusion rate by 65% in resting cells harvested during exponential growth. Higher alcohol concentrations further increased the acetic acid diffusion rate. We demonstrated that facilitated diffusion of acetic acid through Fps1 did not occur under the experimental conditions employed and consequently the acetic acid diffusion measured in our study was passive diffusion across the lipid bilayer of the cell membrane. Passive diffusion is the most relevant mode of acetic acid entry into the cell under industrial conditions, as the adaptation of cells to acetic acid during cell propagation induces endocytosis and degradation of Fps1 4. The observed effects of ethanol on the acetic acid diffusion rate correlated well with the effects reported previously 27 while, to the best of our knowledge, no detailed information on the effect of n-butanol was available prior to this work.

To understand the implications of an increase in acetic acid diffusion rate, caused by alcohols, on acetic acid tolerance, we evaluated the tolerance of *S. cerevisiae* in terms of growth performance in the presence of alcohols and acetic acid. The effect of an increase in acetic acid diffusion rate is probably not proportional to the effect on cell growth in the presence of acetic acid throughout the concentration range, as the effect on growth also depends on the cell’s capacity to extrude acetate and protons. The effect of alcohols on cell growth in the presence of acetic acid was evaluated at 3 g/L and 6 g/L acetic acid. Interestingly, at both the acetic acid concentrations evaluated, cells were more affected by ethanol than by n-butanol when comparing 40 g/L ethanol and 8 g/L n butanol, in spite of that these alcohol concentrations affected the rate of acetic acid diffusion and cell growth to similar extents. For both 40 g/L ethanol and 8 g/L n-butanol, it can be expected that the energy needed for acetate and proton efflux will have increased comparably, due to the similar increase in acetic acid diffusion rate. However, our simulations suggest that ethanol is present both in the membrane and inside the cell, while n butanol would mainly partition in the membrane. Therefore, the stronger effect of ethanol than n butanol is probably not related to the effect on the membrane, but is rather the consequence of ethanol also acting intracellularly, and similar targets of ethanol and acetic acid inside the cell may cause this observed synergistic effect. Indeed, a major reason for cell inhibition by intracellular ethanol is reduced water activity, and intracellular acetic acid combined with ethanol will then further reduce the water activity, possibly below the critical level for cell growth 28.

The biophysical mechanisms behind the effect of ethanol and n-butanol on the acetic acid diffusion rate were elucidated using molecular dynamics simulations of a model membrane designed to resemble the lipid composition of *S. cerevisiae*. To the best of our knowledge, this is the first time biophysical mechanisms governing the effect of ethanol and n-butanol have been elucidated using a complex model membrane with physiological concentrations of the lipids, including IPC, a yeast sphingolipid proven to strongly influence membrane properties 10. In previous studies, membranes with only one or two different lipid species have mainly been used 23,29,30. The complex membrane composition used in the present work enabled us to better approximate the effect of alcohols on the heterogeneous cell membrane of *S. cerevisiae*, as the specific lipid composition has been demonstrated to strongly influence the effect of alcohols 29,30,31. Our simulations showed that alcohols preferably partition into the head group region of the lipid bilayer, a fact also demonstrated in simpler membrane systems 23,30. The stronger effect of n butanol than of ethanol on the acetic acid diffusion rate seems to be due to two distinct biophysical mechanisms. Firstly, n-butanol is more hydrophobic than ethanol, and this difference affects the alcohol partitioning in the membrane: on average 94 ± 5% of the n-butanol molecules were found in the membrane, but only 59 ± 4% of the ethanol molecules. Secondly, n-butanol partitions deeper into the membrane than ethanol, as a direct effect of the longer n-butanol molecule, thereby disrupting more of the van der Waals forces that connect the membrane lipids. Studies on model membranes with only one lipid component confirm that n-butanol partitions into the membrane to a greater extent than ethanol 23,32. However, the fraction of alcohols in the membrane in our simulations is greater than expected from the membrane/water partition coefficients for ethanol and n-butanol, respectively, as measured in a model liposome 33.

The partitioning of alcohols into the head group region of the lipid bilayer pushed the lipids apart, causing an increase in the membrane area. The effect of increased membrane area was much stronger with n butanol than with ethanol, due to both the higher membrane partitioning, and the deeper penetration of the longer n-butanol molecules in the membrane. A direct effect of increased lateral area is decreased membrane thickness, as the lipid tails are given more space to adopt less straight conformations. We observed a reduction in membrane thickness with both alcohols, but not of the same magnitude as the increase in membrane area. With the equal amounts of the two alcohols in the membrane, the effect of n butanol was no stronger than that of ethanol, suggesting that the deeper membrane partitioning of n-butanol did not further affect the membrane thickness. Few previous studies have compared the effect of ethanol and n butanol on the membrane properties, and most of the data available have focused on the effect of ethanol. The increase in area per lipid and the decrease in membrane thickness have been confirmed for ethanol in simpler membrane systems 29,34. The larger increase in area per lipid with n butanol than with ethanol has been confirmed experimentally using membrane vesicles 32, while a computational study indicated a weaker effect of n-butanol 23. However, it is difficult to draw any general conclusions, as the highest n butanol concentration in the latter study was low, 0.3 n-butanol molecules per lipid, and the membrane consisted of only one lipid species. Increased membrane fluidity has been reported as an effect of alcohols in yeast and bacteria 22,35,36, and is a natural consequence of increased membrane area and reduced membrane thickness. In our simulations we determined the lipid order, i.e., the inverse of membrane fluidity, as an average over the lipid chain, and were able to demonstrate a significant reduction in lipid order only at the highest alcohol concentrations evaluated, partly due to large variations in the simulation. Previous studies using molecular dynamics simulations have also shown that relatively high alcohol concentrations are required to perturb membrane fluidity 23,30. It has also been found that microorganisms exposed to alcohol regulate the lipid profile of the cell membrane so as to alter membrane fluidity 22, and high temperature increasing membrane fluidity, combined with lignocellulose derived inhibitors intensified cell inhibition by ethanol 37.

The biophysical mechanisms governing the effects of alcohols on the cell membrane were mainly increased membrane area and, to a smaller extent, reduced membrane thickness and reduced lipid order. These changes in physiochemical membrane properties probably caused the increase in acetic acid diffusion rate that we observed experimentally in *S. cerevisiae. *To obtain further evidence that the changes in physiochemical membrane properties caused by alcohols increase acetic acid membrane diffusion, we determined the number of water molecules in the membrane interior of our simulated model membrane. It was indeed found that the number of water molecules in the membrane was greatly increased upon exposure to alcohols, although 80 g/L ethanol was needed to obtain a significant effect, while only 24 g/L n-butanol was needed. This increased polarity deeper in the membrane, caused by water, probably facilitates the diffusion of acetic acid through the membrane. Previous experiments in a mutant of *S. cerevisiae *lacking aquaporins, and only able to transport water by passive diffusion through the lipid bilayer of the cell membrane, demonstrated an increased rate of water diffusion in cells exposed to ethanol 38, in line with our computational observation of deeper penetration of water molecules in the membrane of cells exposed to ethanol or n-butanol.

This study clearly demonstrates that ethanol and n-butanol partition in the membrane, altering the membrane properties, and providing a likely explanation for the inhibitory effects of ethanol and n butanol. The concentrations of the alcohols evaluated in our experiments are relevant from an industrial perspective, as 40 g/L ethanol has been defined as the minimal titer for an economically feasible process using lignocellulose as raw material 39,40. Furthermore, our work shows that the diffusion rate of acetic acid can be greatly influenced by compounds that partition into the cell membrane, such as alcohols. It also highlights the need for a holistic view of the process and knowledge concerning the parameters influencing the physiochemical properties of the membrane, to support conscious choices in process design and strain engineering. For example, when selecting the appropriate lignocellulosic raw material, it should be borne in mind that softwood has a lower acetyl content than hardwood and annual plants 11, and is perhaps more suitable for alcohol production, while hardwood and annual plants, with a high acetyl content, are better suited as raw materials for products that do not affect the physiochemical properties of the membrane leading to an increased acetic acid diffusion rate.

## Conclusions

Ethanol and n-butanol severely influenced the rate of acetic acid diffusion in *S. cerevisiae. *The increase in diffusion rate was explained biophysically by alcohol partitioning into the head group region of the lipid bilayer, thereby causing a considerable increase in the area per lipid, together with reduced membrane thickness and lipid order. n-butanol affected the acetic acid diffusion rate and growth of *S. cerevisiae* at lower concentrations than ethanol, due to greater and deeper membrane partitioning. The increased acetic acid diffusion rate led to reduced specific growth rates and prolonged lag phase of *S. cerevisiae* in the presence of alcohols and acetic acid; ethanol demonstrating a synergistic effect, while the effect of n-butanol was only additive. The findings of this study demonstrate the successful use of a complex model membrane, including glycerophospholipids, sphingolipids, and sterols, in molecular dynamics simulations, to provide molecular details of the effect of alcohols on the membrane. It further demonstrates that the diffusion rate of acetic acid can be strongly affected by compounds that partition into the cell membrane, and highlights the need for considering interaction effects between compounds in the design of microbial processes.

## MATERIALS AND METHODS

### Strains and cultivation media

*S. cerevisiae* strain CEN.PK 113_7D (*MATa, SUC2, MAL2-8^c^,* Scientific Research and Development GmbH, Germany) was used in all experiments in this study, except for the experiment investigating the effect of Fps1, where the wild-type *S. cerevisiae* strain BY4741 was compared with the mutant strain *S. cerevisiae* BY4741 *fps1*Δ (kindly provided by Professor Stefan Hohmann, Department of Biology and Bio-logical Engineering, Chalmers University of Technology, Sweden). CEN.PK 113_7D cells were cultured in mineral medium (20 g/L glucose, 5 g/L (NH_4_)_2_SO_4_, 0.5 g/L MgSO_4_(7H_2_O, 3 g/L KH_2_PO_4_, 1 mL/L vitamin solution, 1 mL/L trace element solution). Vitamin solution and trace element solution were prepared as described previously 41. Potassium hydrogen phthalate buffer (100 mM) was used to maintain the culture at pH 5. The BY4741 wild-type and *fps1*Δ strains were cultured in YPD medium (20 g/L peptone, 10 g/L yeast extract, 20 g/L glucose) adjusted to pH 5 using KOH, and buffered with 50 mM potassium hydrogen phthalate.

### Medium supplements

Ethanol at concentrations of 40 - 80 g/L, n butanol at concentrations of 8 - 16 g/L and acetic acid at concentrations of 3 - 6 g/L were added to cell cultures to evaluate their effect on the maximum specific growth rate. Stock solutions of 600 g/L ethanol, 37.5 g/L n-butanol and 90 g/L acetic acid were prepared in water and adjusted to pH 5. However, mixing medium and stock solutions each with a pH of 5 did not result in a medium of pH 5 when using highly concentrated stock solutions. Therefore, to ensure the correct pH, potassium hydrogen phthalate buffer was prepared at pH 4, mixed with mineral medium and supplements at pH 5, and the mixtures were then separately adjusted to pH 5 using KOH.

### Inoculum

Inoculum was prepared in Erlenmeyer flasks where the culture occupied a maximum of 10% of the flask volume. Cultures were grown under continuous shaking at 200 rpm, at 30 °C overnight. Exponentially growing cells with an optical density (OD) at 600 nm of 2 were harvested and used to inoculate cultures for acetic acid diffusion rate determination, or to inoculate microscale cultures to determine the maximum specific growth rate in the presence of ethanol, n-butanol and acetic acid.

### Cell cultures for acetic acid diffusion measurements 

Cell cultures of 50 mL in 500 mL Erlenmeyer flasks were inoculated to give a starting OD of 0.1 using an exponentially growing inoculum of OD 2. Cultures were harvested in the exponential growth phase at OD 1, by centrifugation at 3000×g, 4 °C, for 5 minutes, and washed twice in 50 mM ice-cold potassium hydrogen phthalate buffer. Cells were resuspended and concentrated 100 times in 50 mM ice-cold potassium hydrogen phthalate buffer at pH 5, and then stored on ice. The cell dry weight was determined in duplicate for each biological replicate by filtering 200 µL cell suspension through dry, pre-weighed 0.45 µm PES membranes (Sartorius Stedim, Aubagne, France) 13.

### Determination of acetic acid diffusion

Acetic acid diffusion was measured by mixing a small amount of [^14^C] acetic acid with a larger fraction of non-labeled acetic acid. To evaluate the influence of acetic acid concentration on the initial rate of acetic acid diffusion into the cell and the intracellular acetic acid accumulation, as well as the effect of Fps1, a final amount of 1 µCi (20 nCi/µL) [1-^14^C] acetic acid (Perkin Elmer, Mechelen, Belgium), corresponding to 0.36 mM acetic acid, was mixed with 0.2, 2, 20, or 200 mM unlabeled acetic acid. This resulted in total acetic acid concentrations of 0.56, 2.4, 20, and 200 mM, with specific activities of 70 100, 16 600, 2 000, and 200 DPM/nmol acetic acid. Each assay was initiated by incubating 60 µL of cells stored on ice with 180 µL of 50 mM potassium hydrogen phthalate buffer at pH 5, in a 30°C water bath for 4 minutes. Acetic acid diffusion was then measured after the addition of 60 µL acetic acid mixture. Samples of 50 µL were withdrawn from the diffusion assay after 15 s, 40 s, 70 s, 5 min and 10 min, and added to 10 mL of an ice-cold stop solution containing only unlabeled acetic acid. The concentration of acetic acid in the stop solution corresponded to the concentration of acetic acid used in the specific diffusion assay. The stop solution containing the cells was rapidly filtered through Whatman GF/C filters (Ø 25 mm, Whatman, Maidstone, UK). The filters were then washed with 10 mL stop solution and placed in vials with 10 mL Emulsifier-Safe^TM^ scintillation liquid (Perkin Elmer, Groningen, the Netherlands) and shaken thoroughly.

### Determination of acetic acid diffusion kinetics

Acetic acid diffusion kinetics was measured using a final amount of 0.2 - 1.4 µCi (4 - 28 nCi/µL) [1-^14^C] acetic acid mixed with 0.2 - 1.4 mM unlabeled acetic acid, resulting in total acetic acid concentrations of 0.27 - 1.9 mM, with a specific activity of 39 300 DPM/nmol. Each assay was initiated by incubating 10 µL of cells stored on ice with 30 µL of 50 mM potassium hydrogen phthalate buffer at pH 5, in a 30°C water bath for 4 minutes. To evaluate the effect of ethanol and n-butanol, potassium hydrogen phthalate buffer was prepared with the alcohol concentration required to obtain the indicated final ethanol (40 - 80 g/L) and n butanol (8 - 16 g/L) concentrations. To ensure the correct pH, all buffer solutions containing ethanol and n-butanol were separately adjusted to pH 5. Acetic acid diffusion was then measured after the addition of 10 µL acetic acid mixture. Cells were incubated in a 30°C water bath for 30 s before the addition of 10 mL ice-cold stop solution of 2 mM unlabeled acetic acid. The stop solution with the cells was then rapidly filtered and treated as described above for the determination of acetic acid diffusion.

### Analysis of intracellular acetic acid concentration

The amount of intracellular acetic acid was determined by measuring the radioactive decay of [^14^C] acetic acid using a liquid scintillation counter (Perkin Elmer, Wallac Guardian 1414). The number of scintillation counts was correlated to the concentration of acetic acid by preparing standards of the acetic acid mixture added directly to the scintillation liquid. Samples were corrected for acetic acid adsorption on the filter and cell surface by subtracting the radioactivity of blanks prepared with the acetic acid mixture and cells added directly to the stop solution, and rapidly processed according to similar procedure as for the samples. Standards and samples were corrected for natural back¬ground radiation. The intracellular acetic acid concentration was calculated assuming a cell volume of 2 µL/mg dry weight 42. The measured radioactive decay was linear in the concentration range evaluated, and no quenching effects from the sample matrix were observed.

### Microscale cultures to evaluate the effect of ethanol, n-butanol and acetic acid on cell growth

Exponentially growing inoculum was harvested at OD 2 by centrifugation at 3000×g at room temperature for 5 minutes. Cells were concentrated to OD 4 by removing half of the supernatant and resuspending the cells in the remaining supernatant. Microscale cultures of 1 mL were then inoculated at OD 0.2. Aerobic cell growth was automatically monitored in 48 well plates (FlowerPlate® B, m2p labs, Baesweiler, Germany) at 30°C using a BioLector (m2p labs). To minimize evaporation, the plates were covered with a gas-permeable sealing foil with an evaporation-reducing layer (m2p labs). Cultures were shaken contin¬uously at 1200 rpm and the cell density was measured optically every 15 min using a filter gain of 20. Optical density values from the BioLector were converted to OD_600_ values using a standard curve.

### Molecular dynamics simulations

The same membrane model was used as in our previous study 10. It consists of 20 IPC (inositol phosphoryl ceramide) and ergosterol molecules (15% each), together with 44 DOPC (1,2-dioleoyl-sn-glycero-3-phosphocholine) and POPI (1-palmitoyl-2-oleoyl-sn-glycero-3-phospho-(1’-myo-inositol) molecules (35% each). The bilayer was neutralized by 64 sodium ions. The membrane was solvated with 5210 water and alcohol molecules using the Packmol program 43. The alcohols were inserted in the water phase at initial concentrations of 8, 12, 16, 24, 40, 60, 80, 120, 200, and 360 g/L, assuming bulk density of the water and alcohols. Lipids, water and alcohols were described with the Stockholm lipids 44, TIP3P 45, and general Amber force fields 46, respectively. The atomic partial charges on the alcohols were determined using the AM1-BCC method 47 with the Antechamber program 48.

The membranes were simulated with molecular dynamics employing Gromacs software 49, version 4.6. The membranes were simulated for 400 ns; the last 100 ns were used for analysis, and snapshots were collected every 10 ps. Atoms were propagated with a 2 fs time step, and all covalent bonds were constrained with LINCS or SETTLE 50. Pressure was maintained at 1 atm using a Parinello-Rahman barostat 51 with a 10 ps coupling constant, controlling the pressure along the membrane normal independently of the membrane plane. The temperature was maintained at 298 K using a Nosé-Hoover thermostat 52,53 with a 0.5 ps coupling constant. Electrostatic interactions were treated with particle-mesh Ewald summation 54 using a 1 nm real-space cut-off. Van der Waals interactions were cut off at 1 nm, but a long-range continuum correction was added.

The membrane area and the uncertainty in this were calculated from the size of the simulation box. The bilayer thickness was defined as the average distance between the peak density of the phosphate groups in the two leaflets. Lipid tail order was calculated from the average deuterium order parameter, which estimates the orientation of each carbon in the fatty acyl chain, using a standard formula 55. The uncertainties of the thickness and order parameters were estimated with block averaging. The number of water molecules in the membrane interior was determined from the intercept of the density of the water oxygen atoms and the density of the IPC carbon tail atoms: the membrane interior was defined as the area where the tail density was greater than the water density. The uncertainty in the intercept, and thus the uncertainty in the number of water molecules in the interior, was estimated by 100 bootstrap samples of the densities. The area of the bulk water in the simulation was determined from the intercept of the density of the water oxygen atoms and the density of the IPC phosphate moiety: the area of the bulk water was determined to be the area of the simulation box where the water density was greater than the phosphate density. The number of alcohols in the bulk and the membrane was estimated from this intercept and the average area of the membrane. The uncertainties in the number of alcohols in either the bulk or the membrane as well as the uncertainty in the location of the peak density of the POPI glycerol moiety, alcohol hydroxyl and alcohol terminal methyl group were determined by a bootstrap procedure, similar to the uncertainty of the number of interior water molecules. Statistically significant differences refer to significance at the 95% confidence level.

## SUPPLEMENTAL MATERIAL

Click here for supplemental data file.

All supplemental data for this article are also available online at http://microbialcell.com/researcharticles/alcohols-enhance-the-rate-of-acetic-acid-diffusion-in-s-cerevisiae-biophysical-mechanisms-and-implications-for-acetic-acid-tolerance/.
