# Supplementary material for: Alcohols enhance the rate of acetic acid diffusion in S. cerevisiae: biophysical mechanisms and implications for acetic acid tolerance
Source: Microb Cell. 2017 Dec 1;5(1):42–55. doi: 10.15698/mic2018.01.609 (PMC5772038; doi:10.15698/mic2018.01.609)
Supplement: Supplementary file 1 [file mic-05-042-s01.pdf]

Supplemental data

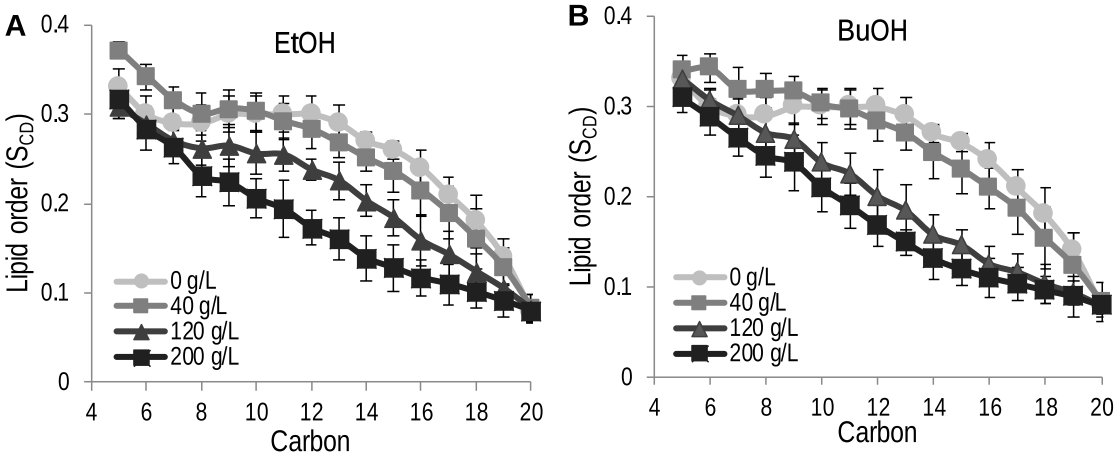

Figure S1: The effect of ethanol and n-butanol on the lipid order of the short chain of IPC. A. Ethanol. **B.** n-butanol. The data shown are the mean over one simulation ± standard deviation.
